# Detection of human annexin A1 as the novel N-terminal tag for separation and purification handle

**DOI:** 10.1186/s12934-022-02005-x

**Published:** 2023-01-05

**Authors:** Xiaomei He, Shuncheng Zhang, Dongya Dang, Tingting Lin, Yuanyuan Ge, Xiaofeng Chen, Jun Fan

**Affiliations:** 1 College of Biological and Pharmaceutical Engineering, West Anhui University, Lu’an, 237012 People’s Republic of China; 2 School of Life Science, Anhui, Anhui Agricultural University, 130, Changjiang West Road, Hefei, 230036 People’s Republic of China

**Keywords:** Annexin A1, Fusion tag, Affinity separation, Affinity purification, Detection, *Escherichia coli*

## Abstract

**Background:**

Several fusion tags for separation handle have been developed, but the fused tag for simply and cheaply separating the target protein is still lacking.

**Results:**

Separation conditions for the human annexin A1 (hanA1) tagged emerald green fluorescent protein (EmGFP) in *Escherichia coli* were optimized via precipitation with calcium chloride (CaCl_2_) and resolubilization with ethylenediamine tetraacetic acid disodium salt (EDTA-Na_2_). The HanA1-EmGFP absorbing with other three affinity matrix was detected, only it was strongly bound to heparin Sepharose. The separation efficiency of the HanA1-EmGFP was comparable with purification efficiency of the His6-tagged HanA1-EmGFP via metal ion affinity chromatography. Three fluorescent proteins for the EmGFP, mCherry red fluorescent protein and flavin-binding cyan-green fluorescent protein LOV from *Chlamydomonas reinhardtii* were used for naked-eye detection of the separation and purification processes, and two colored proteins including a red protein for a *Vitreoscilla* hemoglobin (Vhb), and a brown protein for maize sirohydrochlorin ferrochelatase (mSF) were used for visualizing the separation process. The added EDTA-Na_2_ disrupted the Fe–S cluster in the mSF, but it showed little impact on heme in Vhb.

**Conclusions:**

The selected five colored proteins were efficient for detecting the applicability of the highly selective hanA1 for fusion separation and purification handle. The fused hanA1 tag will be potentially used for simple and cheap affinity separation of the target proteins in industry and diagnosis.

**Supplementary Information:**

The online version contains supplementary material available at 10.1186/s12934-022-02005-x.

## Introduction

Application of proteins and enzymes requires efficient purification process. Several affinity tags comprising His6-tag, maltose binding protein (MBP) and glutathione *S*-transferase (GST) are commonly used for rapid purification of recombinant protein [1]. For increasing protein purity, affinity-purified protein is recovered in a suitable buffer for the subsequent downstream of gel-filtration and/or ion-exchange chromatography. The tandem affinity tag is designed for efficient purifying fusion protein by use of two-step purification strategy [1]. Protein of interest is eluted from the affinity matrix under mild conditions. In most cases, reagents for MBP and GST tag elution display no apparent inhibitory effect of target enzymes.

Except for the well-known tag for affinity chromatography, other fusion partners are used to non-chromatographic purification of target protein. Elastin-like polypeptide (ELP) tag with various repetitions of the pentapeptide VPGXG (10⁓140) presents the revisable aggregation with cycles of salt addition or heating, and centrifugation for obtaining the purified protein [2], but high concentrations of NaCl is usually required for insoluble aggregates formation. Additionally, most proteins are unstable at relatively high temperature. Hydrophobin fusions are easily purified using the aqueous two-phase system, since this protein is self-assembled at hydrophilic-hydrophobic interfaces [3]. However, hydrophobins are often produced as inclusion bodies in *E. coli* [4, 5]. For preparation, the added surfactant is expensive, and isopropyl alcohol probably causes target protein denaturation. A pH-responsive CspB tag only extracellularly produced in *Corynebacterium glutamicum* is designed [6]. Nonetheless, addition of acid for protein precipitation frequently results in denaturation of the fused target protein. So, this tag is only utilized for purifying a few of peptides. Recently, the Ca^2+^ dependent phase-transition properties of calsequestrin (CSQ) permit the protein fused with the Z domain of protein A (ZZ-CSQ) for affinity separation [7]. Developing the novel fusion tag at the N-terminal partner with high selectivity for simple, cost-effective separation and purification is required for isolating recombinant protein and enzyme with industrial and clinical values.

Annexins, an evolutionary conserved multigene family with calcium ion and phospholipid-binding properties, are widely distributed in eukaryotes [8]. In the presence of calcium ion at relatively high concentration, the recombinant annexins in *E. coli* is prone to be aggregated, which can be purified by use of CaCl_2_ for triggering insoluble aggregation, followed by redissolution with calcium chelator ethylene glycol tetra-acetic acid (EGTA) or EDTA [9, 10]. In the presence of calcium ion at low concentration, annexin family members are purified by phenyl Sepharose [11, 12], heparin sepharose [13, 14], and phosphatidylserine covalently coupled to the resin [15]. A few of annexin proteins interact with the S-100 protein [16, 17]. When *Mycobacterium xenopi* GyrA intein is placed between the N-terminal target protein and C-terminal annexin B1 from *Cysticercus cellulosae*, higher purity of the released proteins including interleukin-2 and urokinase are prepared by two cycles of the precipitation/resolubilization process and followed by size-exclusion chromatography [18]. However, incorporation of the intein sometimes showed several disadvantages [19], and annexin as the affinity tag is not well systemically investigated.

The β-barrel based fluorescent proteins such as the GFP variants with improved folding or its color variants with altered excitation and emission spectral properties are widely applied in microbes [20]. The flavin mononucleotide-based fluorescent protein, belonging to a highly conserved family of photoreceptors known as light, oxygen, and voltage (LOV) sensing proteins, possess near-infrared fluorescence, independence of oxygen, small size, and photosensitizer activity. The unique characteristics over the GFPs enable the LOV to be applied for monitoring gene expression, protein solubility, interaction, and transportation in microbes, especially in anaerobes [21]. Vhb is often used in the field of metabolic engineering for microorganisms, plants, and animals, due to enhancement of cell growth, product synthesis and stress tolerance [22]. Siroheme, as the member of tetrapyrroles comprising heme, chlorophyll, and vitamin B_12_, is the cofactor of sulphite and nitrite reductases. The synthetic pathway of siroheme is elucidated, and sirohydrochlorin ferrochelatase (SF) is the terminal enzyme [23]. The SF from Arabidopsis containing the 2Fe-2S center displays brown under the visible light [24]. Our previous study identifies that use of the colored proteins including the EmGFP, mCherry, Vhb and mSF is efficient for visualizing the cellulose-binding module (CBM) tag bound to the regenerated amorphous cellulose (RAC) matrix [25], but naked-eye detection of the protein separation using the fluorescent and colored proteins is not known.

In this study, we analyzed human annexin A1 (hanA1) as the fusion tag for optimizing the separating conditions, observed separation process of the tagged three fluorescent proteins and two colored proteins, analyzed four affinity matrix for purifying the hanA1-EmGFP in crude extracts or via affinity separation, identified the added EDTA-Na_2_ effects on structure of the colored proteins and assayed solubility efficiency of the selected fusion constructs with the added EDTA-Na_2_. The results indicated that the hanA1 tag was used for simple, rapid and cheap separation of the recombinant proteins in *E. coli*.

## Materials and methods

### Plasmids, *E. coli* strains, and reagents

*E. coli* strains DH5α and BL21 (DE3), and the plasmid pET-22b and pET-28b are products of Novagen (USA). Human cDNA library was supplied by Maikun Teng, University of Science and Technology of China. The genes encoding the EmGFP, mCherry, the codon-optimized Vhb, the mature mSF (V71-S212) with insertion at *Bam*H I and *Xho* I sites in the expression vectors, and the helper plasmid for coexpressing yeast mature 5-aminolevulinic acid synthase (ALAS) were constructed by our laboratory [24, 25].

Heparin Sepharose CL-6B, Phenyl Sepharose CL-4B and amylose resin were obtained from GE (Healthcare, USA). Nickel-nitrilotriacetic acid (Ni–NTA) agarose was purchased from Qiagen (Hilden, Germany). Ultra-15 centrifugal filter tubes equipped with the Ultracel-10 membrane were purchased from Merck-Sigma-Aldrich (Kenilworth, NJ, USA). Primers and the gene encoding the CrLOV were synthesized in General Biol Company (Chuzhou, Anhui, China). Monoclonal antibody including mouse anti-GFP, and anti-MBP, and horseradish peroxidase (HRP) conjugated goat anti-mouse IgG antibody, reagents for Western blot analysis were bought from Transgen Biotech (Beijing, China). RAC was prepared from microcrystalline cellulose, according to our previous report [24].

### Plasmids construction

The sequence encoding the hanA1 was amplified by PCR using human brain cDNA library as template, the forward primer CAT CTA CAT ATG GTA TCA GAA TTC CTC AAG CAG and reverse primer GCA GGA TCC GTT TCC TCC ACA AAG AGC CAC CAG. The gel-purified products were digested with *Nde* I and *Bam*H I, and inserted into *Nde* I/*Bgl* II sites of our constructed pCBM-GFP plasmid [25], to generate the resultant vector pA-EmGFP. The similar construction was conducted for the plasmids expressing the hanA1 tagged other target proteins. The fragment encoding the hanA1 tagged EmGFP, Vhb or mSF was excised with *Nde* I and *Xho* I, and subcloned into *Nde* I/*Xho* I sites of the pET-28b vector to express the His6-tagged recombinant constructs. The hS100A11 coding sequence was amplified by using human cDNA library as the template, and forward primer CAT GGA TCC GCA AAA ATC TCC AGC CCT ACA GAG and reverse primer GAT CTC GAG TTA GGT CCG CTT CTG GGA AGG GAC. The PCR products were treated with *Bam*H I and *Xho* I, and inserted into the *Bam*H I-*Xho* I site of the pCBM-GFP plasmid for expressing the CBM-hS100A11, or into the same sites of the pMBP-eDAL plasmid [26] to overexpress the MBP-hS100A11. Based on the characterized LOV from *C. reinhardtii* (CrLOV) amino acid sequence [27], the coding sequence with the preferred *E. coli* codons was synthesized (Additional file 1: Fig. S1), and also subcloned into the *Bam*H I/*Xho* I sites of pA-EmGFP plasmid. All constructs were sequenced to identify the insert correction.

### Detection of the hanA1 tagged EmGFP in *E. coli*

The transformed *E. coli* BL21(DE3) cells were cultured overnight at 37 °C in lysogeny broth (LB, yeast extract 5 g/L, tryptone 10 g/L, NaCl, 10 g/L) with supply of 40 μg/mL kanamycin and diluted to 200 folds. When optical density at 600 nm (OD_600_) of cells was reached about 0.5 as measured on a U-2900 ultraviolet–visible spectrometer (Hitachi, Tokyo, Japan), the fusion protein was induced by use of 0.5 mM isopropyl-β-D-thiogalactopyranoside (IPTG) at 28 °C for 12 h. After centrifugation, cells were re-suspended in buffer A (20 mM Tris–HCl, pH 8.0, 10% glycerol), and photographed under the fluorescent microscope (Olympus, Japan) with the excitation peak 488 nm.

### Separation of the hanA1-EmGFP under different conditions

Cells were washed with buffer A, and disrupted by sonication at 4 °C. After centrifugation at 13,000*g* for 20 min, the target protein was precipitated by addition of CaCl_2_ at different concentrations. The precipitant was dissolved in 10 mL buffer A containing EDTA-Na_2_ at higher concentrations over CaCl_2_, shaken at 100 rpm for 30 min, and centrifuged. The supernatant was concentrated with Ultra-15 centrifugal filter tube and exchanged with buffer A. The fluorescence signal for the EmGFP was measured in a F-4500 fluorescence spectrometer (Hitachi, Japan) with excitation and emission wavelengths at 488 nm and 515 nm [28].

### Protein analysis

Protein amounts in the extracts were determined by Bradford method, using bovine serum albumin as the standard. Proteins samples were analyzed by SDS-PAGE. The gels were captured with Kodak digital camera, and purity of the target protein was quantified with Image-Quant TL software. For Western blot analysis, the fusion protein was analyzed by SDS-PAGE and transferred to polyvinylidene fluoride membrane, immunoblotted, and treated with the anti-GFP antibodies diluted to 5000 folds and HRP conjugated secondary antibodies diluted to 5000 folds as well. The bands were appeared and photographed by adding 0.08% hydrogen peroxide and 4-chloro-1-naphthol solution (dissolved in 20% methanol). For observation the fluorescence emitted from the fusion protein on the gel, protein samples were mixed with SDS-PAGE sample buffer, incubated at 45 °C for 10 min, centrifuged briefly, and separated by SDS-PAGE. The gel was later visualized under UV light and photographed.

### Visualization of affinity separation process using different colored proteins

The hanA1 tagged EmGFP, mCherry, CrLOV, and mSF were produced as the reported induction conditions [25]. The supernatants were added CaCl_2_ at 10 mM (final concentration), and re-solubilized protein with supplemented 15 mM EDTA-Na_2_ (final concentration). The separated and solubilized proteins were photographed under UV or visible light. The His6-tagged mSF was purified by Ni–NTA, and the eluted protein with buffer A exchange was analyzed on the spectrometer for recording UV–Vis absorption spectra at 300–700 nm. For the purified His6-hanA1 tagged mSF, spectra in the presence of EDTA-Na_2_ was recorded. The Fe-S cluster in Arabidopsis is reconstituted under strictly anaerobic condition, by incubation of mixture containing Fe(NH_4_)_2_(SO_4_)_2_ and Na_2_S with the purified SF prior to 2% (w/v) 2-mercaptoethanol for 1 h [24]. In this work, we performed aerobic reconstitution of Fe-S cluster in the mSF incubated with the same compounds. The chelating agent was also added to the prepared solution containing the heme-bound Vhb, and the spectra at 250–500 nm was scanned. To examine solubilzation efficiency with the added EDTA-Na_2_, two equal volumes of protein from clear lysates in tubes with the same concentration (1 mg/mL) were precipitated by adding 10 mM CaCl_2_. After the mixture was centrifuged at 12,000 g for 10 min at room temperature, and washed twice with buffer A containing 10 mM CaCl_2_, the precipitants on one tube were solubilized by addition of 8 M urea. With removal of precipitants at the same centrifugation condition, protein amounts were measured by Bradford method. For another tube, 10 mM CaCl_2_ (final concentration) was added and mixture was centrifuged. The soluble proteins in the supernatant was examined.

### Affinity purification

The hanA1-EmGFP proteins from soluble extracts or through affinity separation were dissolved in buffer B (buffer A containing 1 mM CaCl_2_), further purified by heparin Sepharose CL-6B (1.0 × 5 cm), based on the published report [20]. The column was washed with buffer B, and the fusion protein was eluted with buffer A containing 5 mM heparin and 5 mM EDTA-Na_2_. For purifying the fusion protein by phenyl Sepharose CL-6B (1.0 × 3 cm) at room temperature [19], the separated fusion protein was loaded on the resin for elution with 30, 20% and 10% saturated ammonium sulfate (AS), respectively. The eluted proteins were analyzed by SDS-PAGE.

For oriented immobilization of the MBP-hS100A11, amylose resin was washed with buffer A containing 250 mM NaCl, followed with buffer B. The immobilized MBP-hS100A11 was used for absorbing with the hanA1-EmGFP in crude extracts or via affinity separation. The bound hanA1-EmGFP was eluted with buffer C (buffer A containing 5 mM EDTA-Na_2_). The increased oriented immobilization of the CBM-hS100A11 on RAC was accomplished, based on the published method [25]. After incubation at room temperature for 3 h, the resin was washed three times with buffer A containing 500 mM NaCl for removal of the contaminated proteins, and re-suspended with buffer B. The hanA1-EmGFP dissolved in buffer B in clear lysate or via affinity separation was incubated with the immobilized MBP-hS100A11 or CBM-hS100A11 at room temperature for 2 h. The resin was washed with buffer B containing 50 mM NaCl, and the target protein was eluted with buffer C.

The His6-hanA1 tagged constructs were loaded on Ni–NTA resin with buffer D (50 mM sodium phosphate, 300 mM NaCl, 10 mM imidazole, pH 8.0), washed with buffer D containing 30 mM imidazole (pH 8.0), and eluted with buffer D containing 250 mM imidazole (pH 8.0). For purified His6-tagged hanA1-EmGFP, the eluent was diluted 10 folds using buffer A, and CaCl_2_ at different concentrations were added. The mixture was centrifuged at 12,000 g for 20 min at room temperature. The precipitated proteins were analyzed by SDS-PAGE, and fluorescence from the supernatant after precipitation was measured.

### Visualization of affinity purification using different fluorescent proteins

Three fluorescent proteins bound to the heparin Sepharose, the immobilized MBP-hS100A11 on amylose resin, or phosphatidylserine were visualized. After incubation, the mixture was centrifuged at 2500 g at room temperature for 10 min. The resin was washed three times with 2 mL buffer B, and incubated with buffer C. The resin and supernatants were photographed under UV and visible light.

### Statistical analysis

Data from three technical replicates were indicated as means ± standard deviations (SD), evaluated by use of a one-tailed t-test, and analyzed using SPSS ver. 22 (SPSS Inc., USA).

## Results

### Evaluation of the added salt affecting CaCl_2_ precipitation of the hanA1-EmGFP

**Fig. 1:**
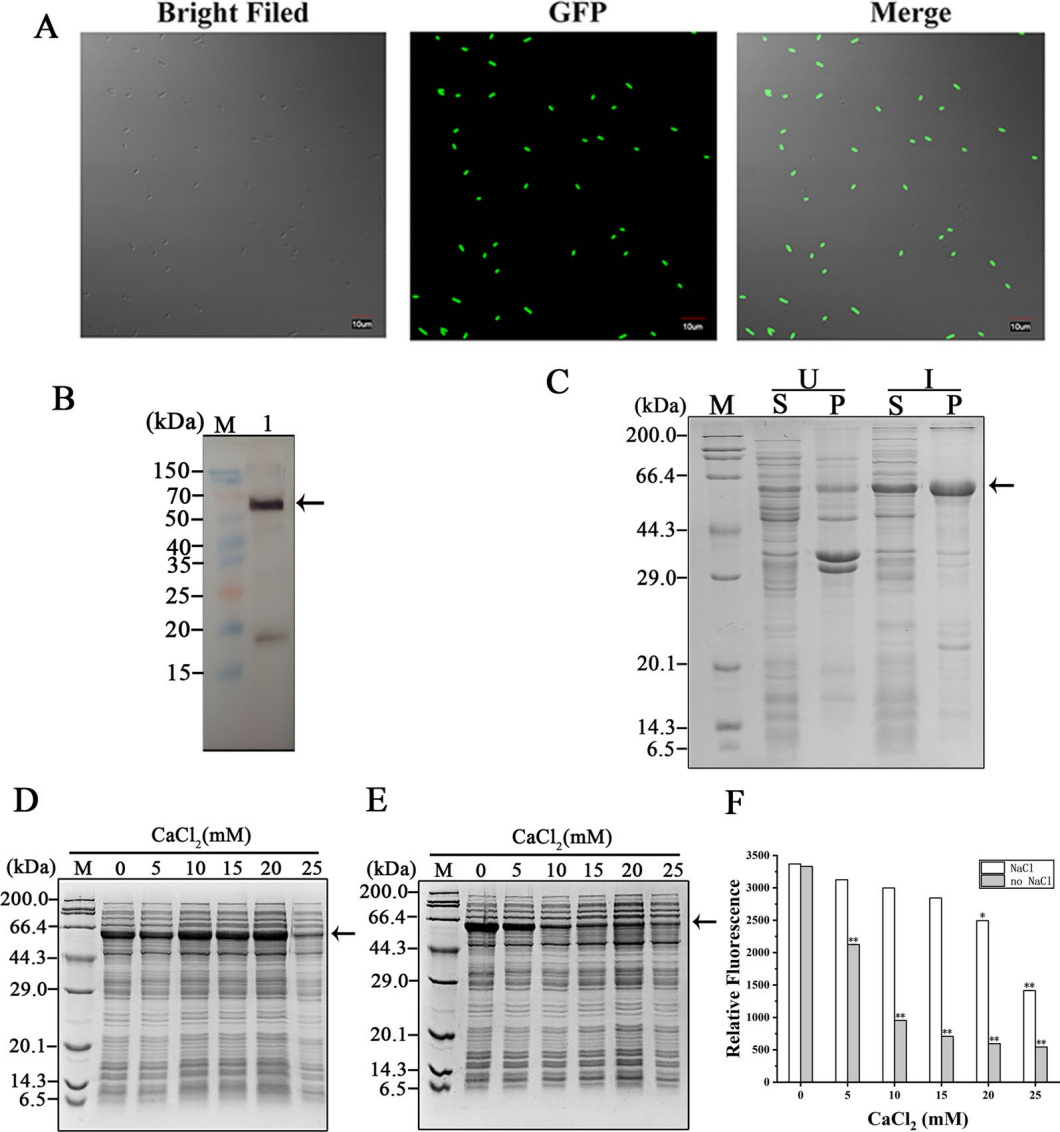
Overexpression and separation of the hanA1-EmGFP. **A** The confocal fluorescent micrographs for *E. coli* BL21(DE3) cells overexpressing the hanA1-EmGFP. Size bars, 10 μm. **B** Western bolt analysis of the induced EmGFP partner using anti-GFP antibodies. In all figures for SDS-PAGE and Western blot analyses, protein marker was labeled as “M”. Lane 1 represented the immunoblot analysis. The arrow indicated the hanA1-EmGFP. **C** SDS-PAGE analysis of the soluble and insoluble hanA1-EmGFP produced in *E. coli* BL21(DE3) cells. U: uninduced. I: induced. S: surpertant. P: pellet. **D** SDS-PAGE analysis of the extracts dissolved in buffer A with 100 mM NaCl substitution of 10% glycerol for precipitation. The final concentrations of added CaCl_2_ were denoted on the top of gels. **E** The correspondent samples dissolved with buffer A were also precipitated with the same treatment. All arrows indicated the bands representing the overexpressed fusion protein. **F** The retained fluorescence from extracts containing the fusion protein in absence and presence of 100 mM NaCl after precipitation treatment. The asterisk indicated significant differences lower than the clear lysate without precipitation as the control; * p < 0.01

In order to detect the hanA1 tag fusion protein solubility, we first used the fused EmGFP as the reporter. This protein shows strong fluorescence and color intensity, compared with the other variant, such as the enhanced GFP (eGFP) variant [28]. The fluorescent *E. coli* cells overexpressing the hanA1-EmGFP were observed (Fig. 1A). The soluble production of the hanA1-EmGFP was detected by Western blotting (Fig. 1B). The fusion protein was produced in *E. coli* as soluble and insoluble forms, as shown on the SDS-PAGE gel (Fig. 1C). Substitution of glycerol in buffer A with 100 mM NaCl decreased the effect of the added 10 mM CaCl_2_, as shown on the SDS-PAGE gels (Fig. 1D, E). With augment of Ca^2+^concentrations, fluorescence in the supernatant was decreased more sharply than that in the presence of 100 mM NaCl (Fig. 1F). The salt is often used to increase solubility of recombinant protein, but it decreased Ca^2+^-dependent aggregation simultaneously.

### Evaluating CaCl_2_ precipitation of the hanA1-EmGFP under two specified temperatures

**Fig. 2:**
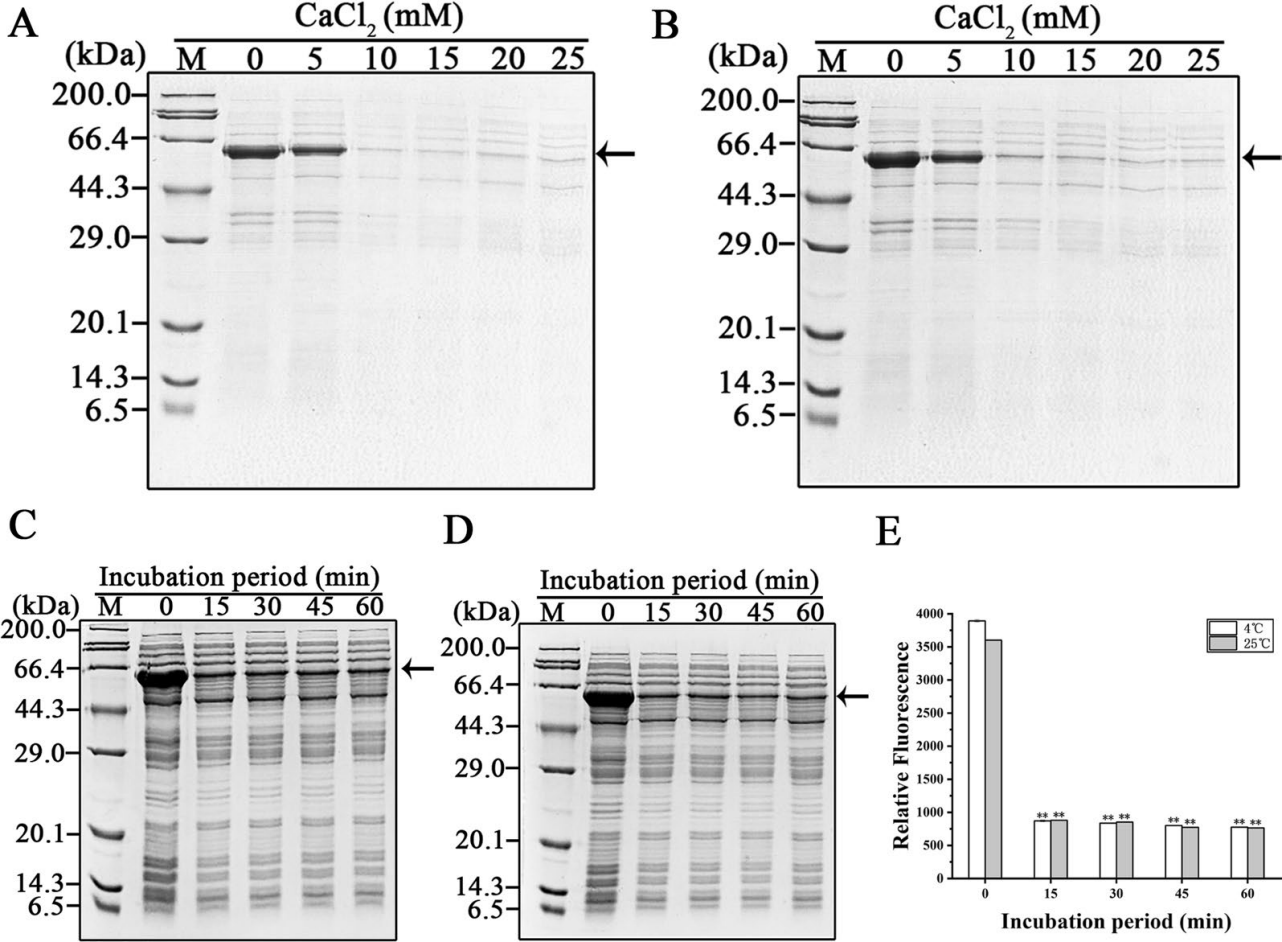
Separating the hanA1-EmGFP from the supernatant at 4 °C and 25 °C. **A** SDS-PAGE analysis of protein samples dissolved in buffer A after CaCl_2_ at different concentrations to precipitate the hanA1-EmGFP at 4 °C. **B** The same analysis for precipitating the fusion protein at 25 °C. **C** SDS-PAGE analysis of the supernatant after adding 10 mM CaCl_2_ for incubating various periods at 4 °C. **D** The same analysis of the correspondent samples for incubation at 25 °C. Arrows indicated the hanA1-EmGFP. **E** The fluorescence from clear lysates containing the fusion protein precipitated with 10 mM CaCl_2_ and stored at different periods at correspondent temperatures. The asterisk in this and other figures indicated significant differences lower than the clear lysate without precipitation as the control; * p < 0.01

Because they are often unstable, proteins were separated, if desired, at low temperature. We precipitated the hanA1-EmGFP in buffer A at 4 °C and 25 °C. Supplementation of 10 mM CaCl_2_ sufficed to precipitate the fusion protein (Fig. 2A, B). The equal amounts of CaCl_2_ exhibited comparable precipitation effect at two different temperatures after incubation over 15 min, as shown on the SDS-PAGE gels (Fig. 2C, D). The fluorescence from the mixture with removal of the precipitants was decreased was almost equivalent after the protein was separated at two specified temperatures (Fig. 2E). Based on the fluorescence measurement, nearly 80% hanA1-EmGFP was precipitated. In contrast, deletion of C-terminal 16 amino acid residues in the ZZ-CSQ allowed about 93% fusion protein to form a precipitate at 10 mM CaCl_2_ within 10 min [7].

### Optimization of CaCl_2_ precipitation and resolubilization of the hanA1-EmGFP

**Fig. 3:**
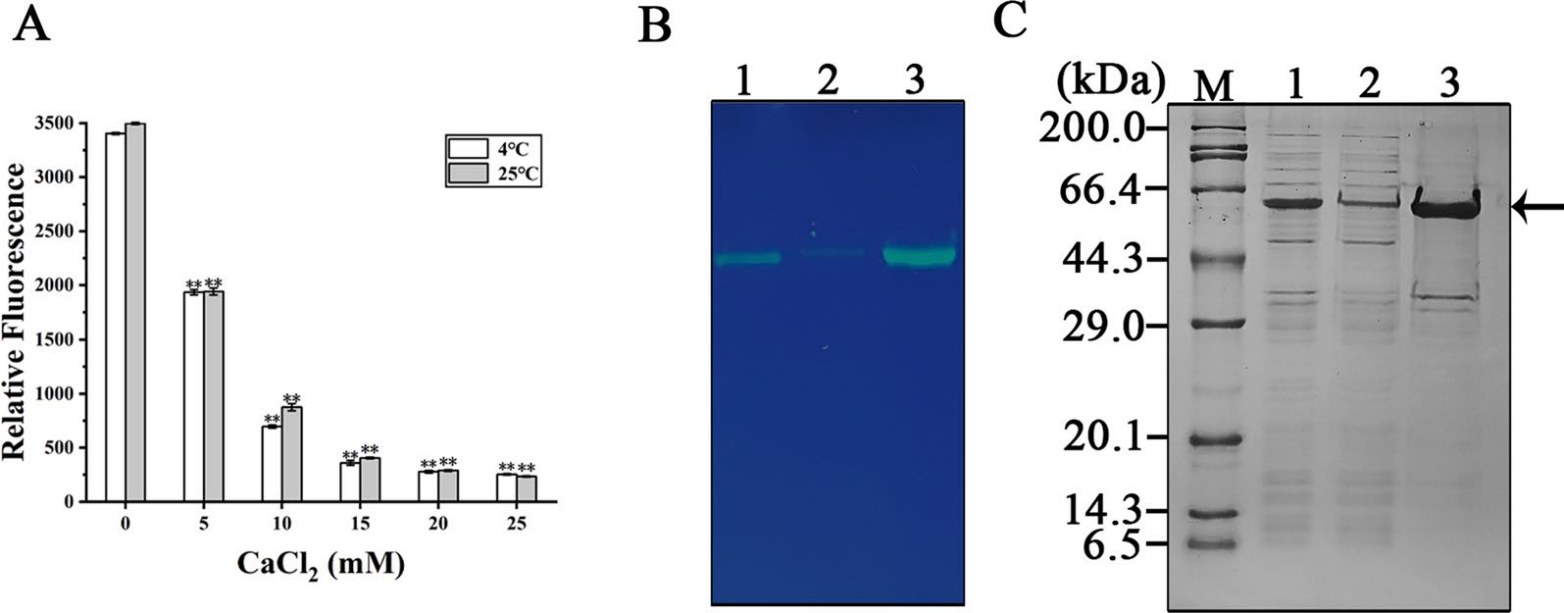
Optimization of the hanA-EmGFP separation method. **A** The fluorescence in soluble fractions in buffer A after CaCl_2_ at different concentrations were added for separation at 4 °C and 25 °C. **B** Fluorescence display on the SDS-PAGE gel for the correspondent protein samples under UV light. Lane 1: soluble fraction. Lane 2: the proteins left in supernatants after 10 mM CaCl_2_ precipitation. Lane 3: the precipitated proteins were re-solubilized with 15 mM EDTA-Na_2_. **C** SDS-PAGE analysis of the correspondent protein samples. The arrow indicated the hanA1-EmGFP

In order to further determine the desirable concentration range of CaCl_2_ for precipitating the hanA1-EmGFP, we separated protein from the clear lysates dissolved in buffer A with the increased CaCl_2_ amounts at 4 °C and 25 °C, and measured the fluorescence without the precipitants. As a result, over 10 mM CaCl_2_ supplementation, the fluorescence was decreased slowly (Fig. 3A). So, we separated the fusion protein using 10 mM CaCl_2_ at 25 °C. In-gel fluorescence of the same amounts of protein samples displayed that, after separation, the hanA1-EmGFP left in the mixture retained relatively weak fluorescence, whereas the EDTA-Na_2_ re-solubilized protein displayed strong fluorescent band (Fig. 3B), in accordance with the fluorescence measurement. The separated fusion protein was not degraded in vivo, and the fusion protein was selectively separated from other contaminating *E. coli* biomolecules (Fig. 3C). The main band representing the hanA1-EmGFP was separated by the first round of the precipitation/resolubization process, but several contaminated protein bands were observed. With two rounds of separation processes, about 7 mg of fusion protein with around 67% purity, was obtained from approximately 27 mg of proteins in soluble extract (Table 1). After two rounds of separation, purity of the fusion protein was further increased, as detected by SDS-PAGE (Additional file 1: Fig. S2). In contrast, the recovery was not decreased significantly (Table 1). The results suggested that N-terminal hanA1 tag was efficient for separation handle.

**Table 1:** Separation efficiency of the hanA1-EmGFP from 200 mL culture ^a^

	Total protein/mg	Purity/%^b^	Yield/mg^c^	Recovery/%^d^
Crude extract	27 ± 4	37 ± 5	10 ± 4	100
First-round separation Second-round separation	15 ± 3 11 ± 2	57 ± 5 67 ± 4	8 ± 3 7 ± 1	83 ± 5 75 ± 4

^a^ Values are averages of experiments performed in triplicate ± standard error

^b^ Determined by densitometric analysis of Coomassie-stained SDS-PAGE

^c^ Yield = total protein × purity

^d^ Recovery is calculated as the residual yield divided by that from the crude extract

### Visualizing separation of the hanA1 tagged colored proteins

**Fig. 4:**
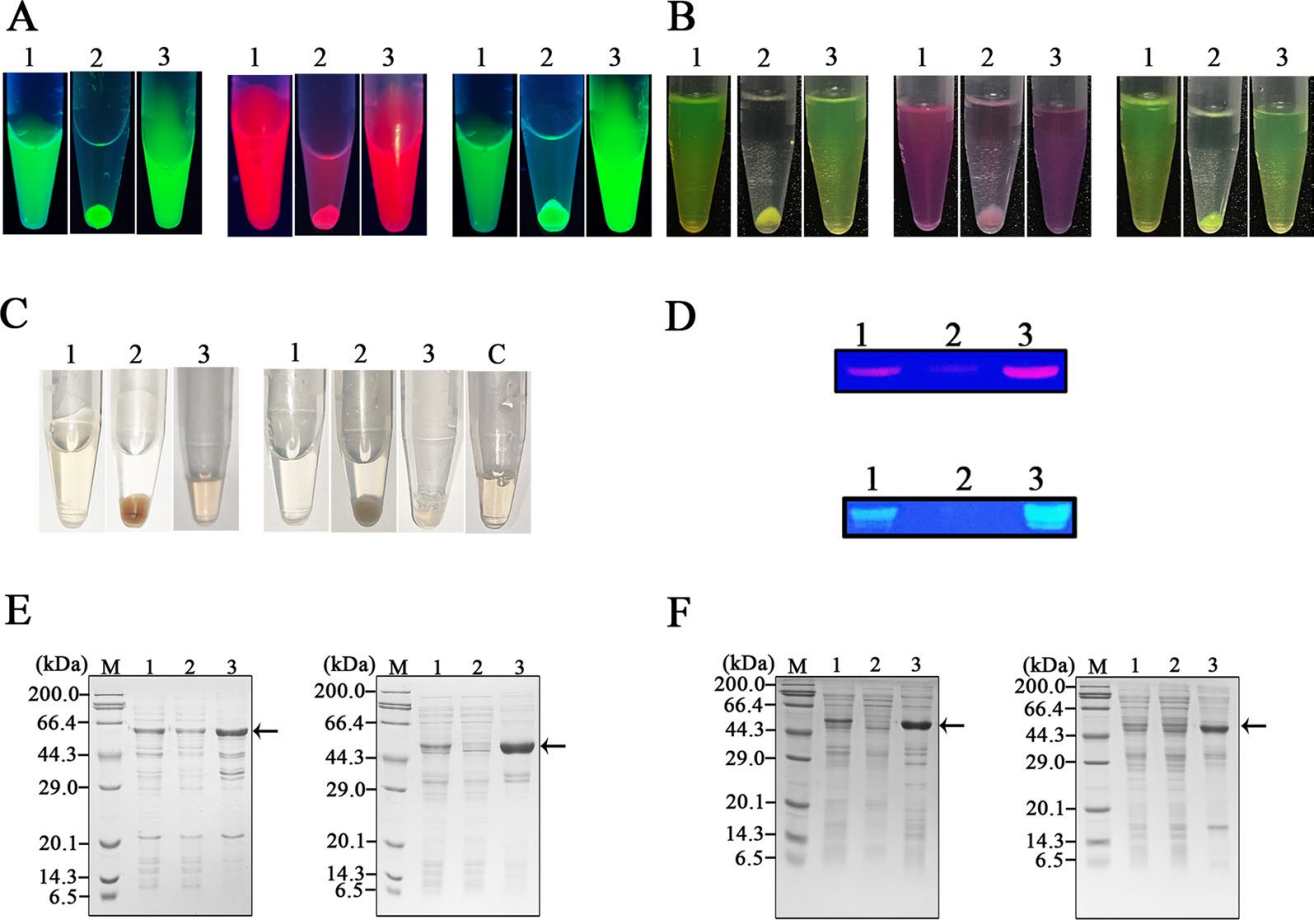
Observation of the colored proteins separated by precipitating and re-solubilizing process. **A** The fluorescent proteins separation observed under UV light irradiation. Left: hanA1-EmGFP. Middle: hanA1-mCHerry. Right: hanA1-CrLOV. In Fig. 4 1: supernatant. 2: supernatant after CaCl_2_ precipitation. 3: supernatant with EDTA-Na_2_ redissolution. **B** The correspondent samples containing the fluorescent protein displayed under the visible light. **C** The colored proteins separation observed under visible light. Left: the hanA1-Vhb. Right: the hanA1-mSF. The purified His6-mSF containing the Fe-S cluster was indicated as C. **D** Fluorescence display of the mCherry (up), and CrLOV (down) with the hanA1 tag on the SDS-PAGE gel. **E** SDS-PAGE analysis of the hanA1-tagged mCherry (left) and CrLOV (right) after separation. **F** SDS-PAGE analysis of the hanA1-tagged Vhb (left) and mSF (right) after separation. In **D** and **E**, Lane 1: soluble fraction. Lane 2: the retained soluble proteins after precipitation. Lane 3: the re-solubilized proteins. Arrows indicated target proteins

The precipitation and re-solubilizition effects were observed for hanA1 tagged three fluorescent proteins including EmGFP, mCherry and CrLOV during preparation under UV irradiation (Fig. 4A) and visible light (Fig. 4B), as well as the tagged two colored proteins including Vhb and mSF under visible light (Fig. 4C). The separated mCherry and CrLOV partners were detected by fluorescence on the SDS-PAGE gel (Fig. 4D), due to the protein resistant to SDS during electrophoresis. SDS-PAGE analysis showed that purities of the mCherry and CrLOV (Fig. 4E), Vhb and mSF (Fig. 4F) as fusion partners were improved by separation. Among the separated proteins, highest amounts of soluble proteins from cells overexpressing the hanA1-Vhb were extracted (Table 2), most likely due to the Vhb responsible for transporting oxygen to stimulate cell growth. The least yield of soluble proteins from cells overexpressing the hanA1-mSF was achieved, probably attributed to cell growth inhibited by addition of Fe(NH_4_)_2_(SO_4_)_2_ to benefit formation of holo-emzyme. The recoveries for all proteins via one round separation were over 70% (Table 2), indicating that the hanA1 tag was utilized for separating other proteins with various protein folding efficiency and solubility.

**Table 2:** Summery of hanA1 tagged other colored proteins for affinity separation ^a^

	mCherry	CrLOV	VHb	mSF
Total protein (mg) ^b^	22 ± 2	19 ± 2	39 ± 4	18 ± 2
Purity (%)	30 ± 3	28 ± 4	15 ± 3	6 ± 3
Yield (mg)^c^	12 ± 1	9 ± 1	12 ± 2	2 ± 1
Purity (%)	44 ± 5	52 ± 3	39 ± 6	35 ± 3
Recovery (%)	79 ± 5	80 ± 4	77 ± 5	70 ± 2

^a^ The purification table was prepared as Table 1

^b^ Proteins were overexpressed in 200 mL LB culture

^c^ All proteins were obtained via one round of separation

### Analysis of the added EDTA-Na_2_ on chromophores in two colored proteins

**Fig. 5:**
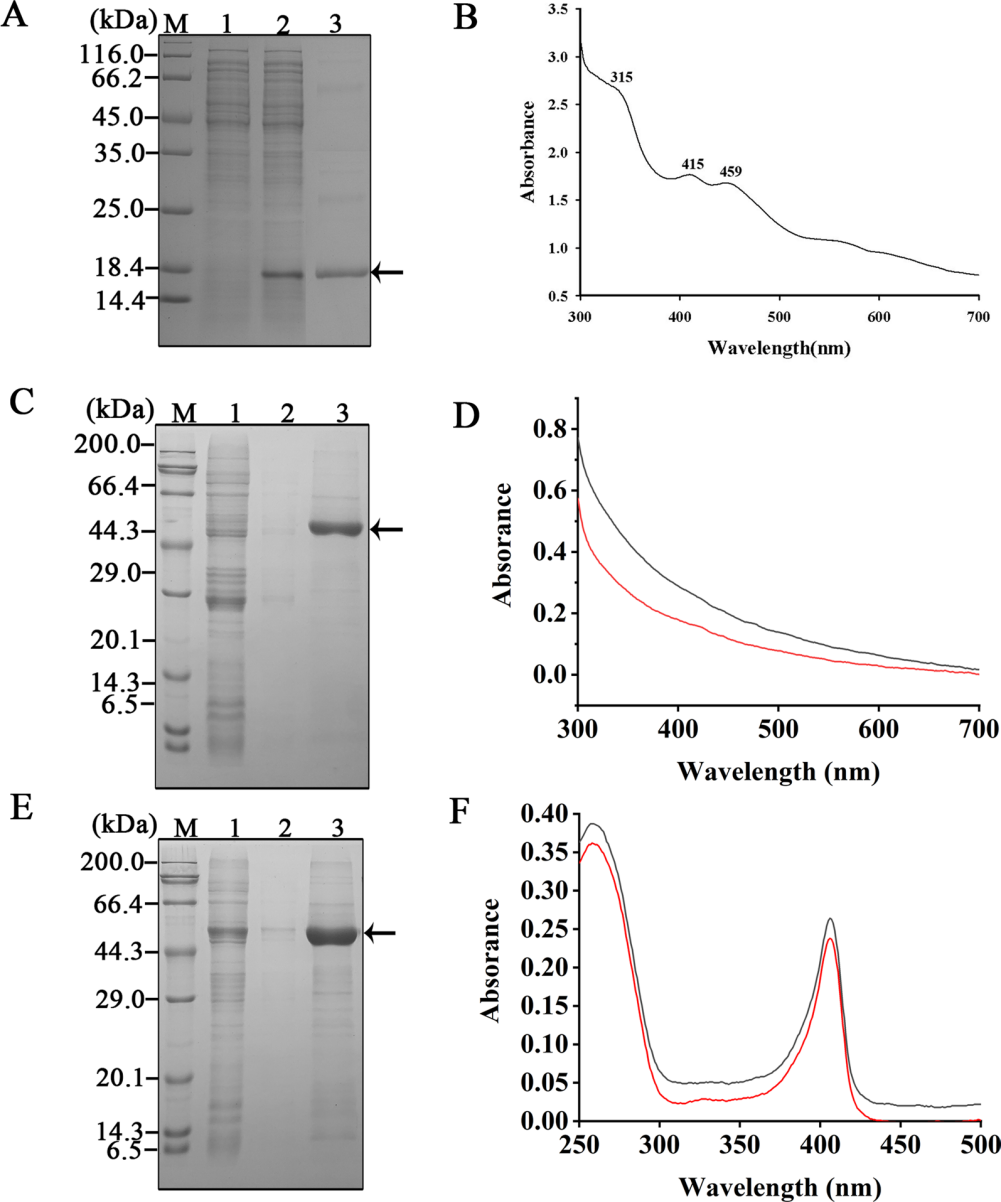
Analysis of the added EDTA-Na_2_ on chromophores in two colored proteins. **A** SDS-PAGE analysis of purified His6-mSF. **B** UV–visible spectrum. The mSF was analyzed at room temperature on the spectrometer using a quartz cuvette sealed with a rubber septum. **C** SDS-PAGE analysis of purified His6-tagged hanA1-mSF. **D** UV–visible spectrum. The purified protein was incubated with 15 mM EDTA-Na_2_, exchanged with buffer A and analyzed at room temperature (black line). After purified protein was incubated with the compounds to reconstitute the Fe-S cluster under aerobic condition, the UV–visible spectrum (red line) was analyzed. **E** SDS-PAGE analysis of purified His6-tagged hanA1-Vhb. **F** UV–visible spectra of the purified hanA1-Vhb in the absence (black line) or the presence of Fe^2+^ (red line) treatment followed by exchange with buffer A. In this figure, lane 1: uninduced proteins. Lane 2: induced proteins. Lane 3: The eluted protein indicated by the arrow. The analyses were described in materials and methods section

Since the separated hanA-mSF contained impurities, we purified the His6-tagged mSF with high purity (Fig. 5A). UV–visible spectra showed absorption peaks at about 342, 415 and 455 nm (Fig. 5B), consistent with that from purified Arabidopsis SF [23]. The His6-hanA1 tagged mSF was also purified by Ni–NTA, and showed the improved purity (Fig. 5C). The added EDTA-Na_2_ resulted in disappearance of 415 and 455 nm peaks, indicating that the Fe–S cluster was partly impaired. Based on UV–visible spectra (Fig. 5D), reconstitution of the Fe-S cluster under aerobic condition was failed. The purified His6-hanA1 tagged Vhb exhibited the enhanced purity (Fig. 5E). The absorption peak was observed (Fig. 5F), agreed with that of the recombinant cytochrome binding heme [29]. Derived from the scanned spectra (Fig. 5F), addition of the chelating reagent hardly impaired heme in the hanA1-Vhb. Our works presented that the colored proteins were applied for visualizing the separation process. The presence of EDTA-Na_2_ affected mSF structure.

### Affinity purification of the hanA1-EmGFP and the bound fluorescent proteins detection

**Fig. 6:**
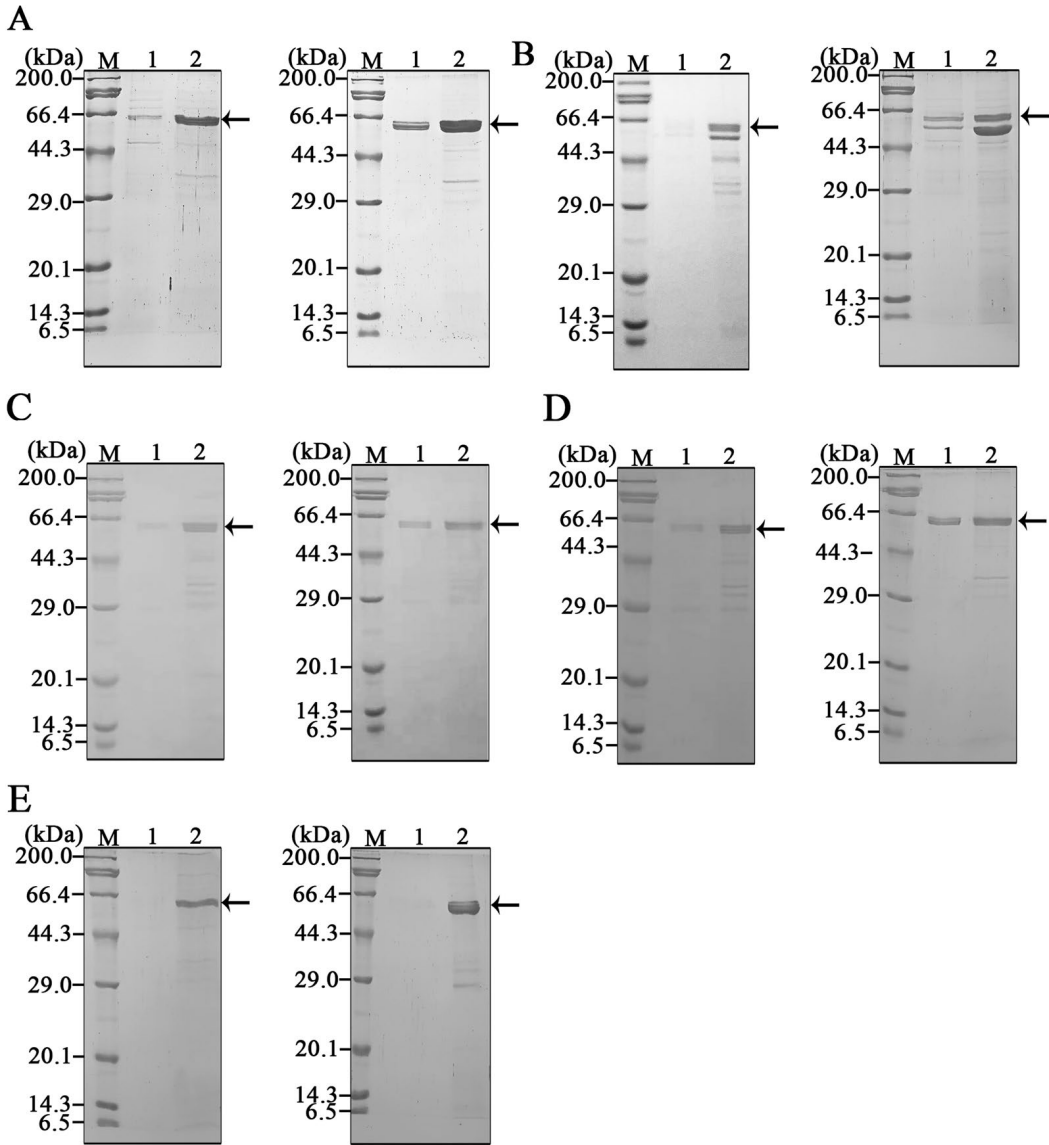
SDS-PAGE analysis of the hanA1-EmGFP constructs via purification. **A** Heparin Sepharose purification. **B** Human S100A11 immobilized on amylose resin through the fused MBP tag at N-terminus for purification of the EmGFP construct. **C** Water-insoluble phosphatidylserine as the affinity matrix for purifying the protein. **D** Phenyl Sepharose purification. **F** Ni–NTA purification of His6-tagged hanA-EmGFP. In each figure, left: direct purification of the protein in soluble extracts. Right: purification of the protein via affinity separation. Lane 1: the washed proteins. Lane 2: the eluted proteins. Arrows indicated the fusion protein

Because annexins are bound to the other affinity resins [10–17], we used the hanA1-EmGFP for purification via other affinity purification directly, or combination of the separation process with affinity purification. The fusion protein was absorbed with heparin Sepharose. The main band on the SDS-PAGE gel represented the purified protein with the increased purity (Fig. 6A, left). Combination of the affinity separation and purification also made fusion protein purity improved, as analyzed by SDS-PAGE (Fig. 6A, right). The MBP-hS100A11 was solubly produced in *E. coli* (Additional file 1: Fig. S3A, lane 1), and immobilized on amylose resin for removing impurities (Additional file 1: Fig. S3A, lane 2). After the hanA1-EmGFP in the crude extract was incubated with the immobilized S100A11, less protein was washed. The MBP-hS100A11 was eluted, but a band closed to the hanA1-EmGFP was shown (Fig. 6B, left). The band was accredited to the co-eluted MBP-hS100A11, as detected by the anti-MBP antibodies (Additional file 1: Fig. S3B). The separated hanA1-EmGFP was co-eluted with the immobilized MBP-hS100A11 as well (Fig. 6B, right). The CBM-hS100A11 strongly bound to RAC resin was also eluted together with the hanA1-EmGFP by use of buffer C (Additional file 1: Fig. S4A and S4B). The hanA1 tagged EmGFP was partly bound to water-insoluble phosphatidylserine as the affinity matrix, and the eluted protein containing impurities (Fig. 6C, left). After separation, the eluted fusion protein showed the enhanced purity (Fig. 6C, right).

The overexpressed or separated hanA1-EmGFP construct was purified by phenyl Sepharose for hydrophobic interaction chromatography (HIC). The fusion protein was eluted upon 10% saturated AS in buffer A, and the eluted protein showed the better purity (Fig. 6D), suggesting that hanA1 was similar to the annexin V with loose interaction with the hydrophobic resin. As comparison, in the absence of CaCl_2_, the fusion protein was eluted from phenyl Sepharose by 30% saturated AS, indicating that Ca^2+^ at the specified concentration impacted the HIC property of the fusion protein. Among the four purification methods, the resin coupled with heparin or phenyl were efficient for higher recovery of the hanA1-EmGFP (Table 3). Covalent coupling of the hS100A11 or phosphatidylserine to the resin will probably enhance the purification efficiency.

**Table 3:** Summery of hanA1-EmGFP for purification via binding the coupled or immobilized molecules ^a^

	Heparin		hS100A11^b^		Phosphatidylserine		Phenyl	
	Sup^c^	Sep^d^	Sup	Sep	Sup	Sep	Sup	Sep
Loaded (mg)	14 ± 2.6	10 ± 2.0	11 ± 2.2	9.3 ± 1.9	12 ± 2.0	9.0 ± 2.0	13 ± 2.1	8.6 ± 1.3
Eluted (mg)	3.9 ± 0.4	5.4 ± 0.3	2.8 ± 0.7	4.5 ± 0.3	1.0 ± 0.1	1.6 ± 0.2	3.9 ± 0.2	4.6 ± 0.2
Purity(%)	80 ± 4.7	86 ± 5.1	42 ± 6.9	44 ± 6.1	72 ± 5.7	91 ± 4.2	88 ± 4.0	91 ± 4.4
Recovery (%)	61 ± 5.0	83 ± 4.7	20 ± 6.3	37 ± 5.6	17 ± 3.5	26 ± 4.5	71 ± 4.5	78 ± 3.8

^a^ The purification table was prepared as Table 1

^b^ The hS100A11 was immobilized through the fused MBP tag on amylose resin

^c^ Sup: supernatant from *E. coli* cells overexpressing the hanA1-EmGFP

^d^ Sep: the hanA1-EmGFP via affinity separation and buffer was exchanged with buffer B

The His6-tagged hanA1-EmGFP was strongly absorbed to Ni–NTA resin, and eluent protein displayed relatively higher purity (Fig. 6E, left). The chimeric protein via affinity separation was also purified by Ni–NTA agarose and eluent showed the fusion protein with relatively high purity (Fig. 6E, right). For three His6-hanA tagged constructs, combining the separation and Ni–NTA purification increased protein purity, but decreased recovery simultaneously (Table 4). Attempt to precipitate the His6-tagged hanA1-EmGFP in the eluent from the Ni–NTA resin for dilution for 10 folds with use of CaCl_2_ at high concentration was unsuccessful, as detected by SDS-PAGE (Additional file 1: Fig. S5A), and fluorescence measurement (Additional file 1: Fig. S5B), due to the presence of 30 mM NaCl and 25 mM imidazole affecting the CaCl_2_ precipitating effect. It is documented that His6-calmodulin (CaM) tagged proteins are eluted and loaded on phenyl Sepharose directly for further removing salts after the proteins were released from the resin by using the buffer containing the EDTA-Na_2_ [30]. Owing to the hanA1 resembling the annexin V with loose interaction with the hydrophobic resin [11], selection of the annexins with strongly bound to phenyl Sepharose [12], is essential to remove high concentrations of salts for eluting the His6-annexin tagged protein from Ni–NTA.

**Table 4:** Summary of the His6-tagged hanA1 fusion constructs purification results by Ni–NTA

	Total protein (mg)	Yield (mg)	Purity (%)	Recovery (%)
EmGFP				
Ni–NTA	22 ± 1	6.0 ± 0.5	87 ± 3	64 ± 2
Separation/Ni–NTA	23 ± 1	4.3 ± 0.3	91 ± 4	45 ± 3
Vhb				
Ni–NTA	26 ± 2	6.7 ± 0.4	89 ± 3	66 ± 2
Separation/Ni–NTA	25 ± 2	5.1 ± 0.2	92 ± 3	53 ± 2
mSF				
Ni–NTA	17 ± 2	1.2 ± 0.1	90 ± 3	61 ± 2
Separation/Ni–NTA	19 ± 2	1.0 ± 0.1	93 ± 3	48 ± 3

### Detection of affinity purification of hanA1 tagged fluorescent proteins

**Fig. 7:**
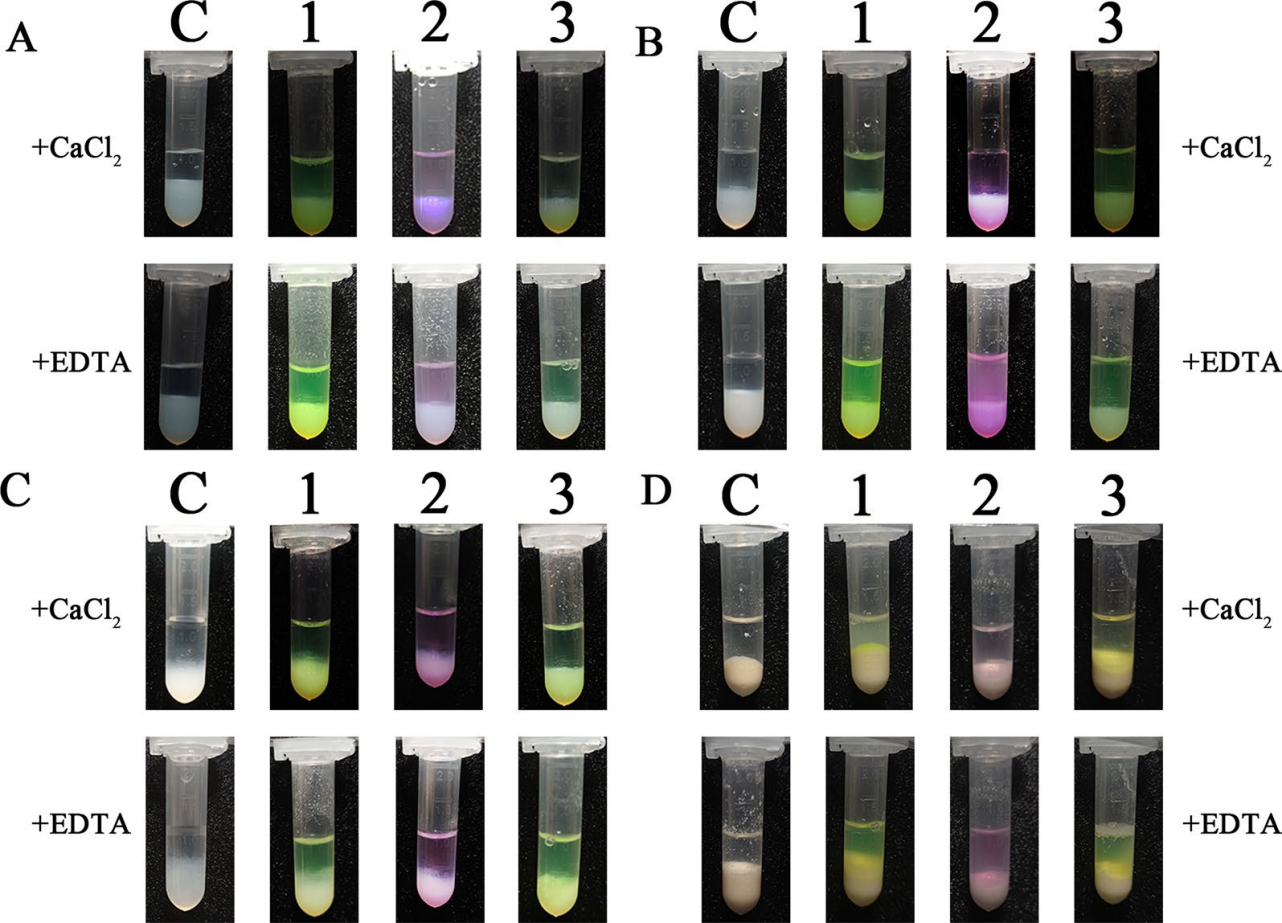
Visualization of the hanA1 tagged fluorescent proteins bound to the affinity resins in buffer B and released from the resin in buffer C. **A** The tagged proteins bound to heparin Sepharose. **B** The tagged proteins released from heparin Sepharose. **C** The tagged proteins bound to the immobilized MBP-hS100A11 on amylose resin. **D** The tagged proteins released from the immobilized MBP-hS100A11. **E**, **F** The tagged proteins were bound to and released from phosphatidylserine. **C** The affinity matrix without binding the proteins. 1: hanA-EmGFP. 2: hanA-mCherry. 3: hanA-CrLOV

The hanA1 tagged EmGFP, mCherry and CrLOV, association and disassociation with the affinity resins were observed under visible light. Three fluorescent proteins were bound to heparin Sepharose in buffer B (Fig. 7A), but was released from the resin in buffer C (Fig. 7B). Analogous association and disassociation of the tagged proteins with immobilized MBP-hS100A11 on amylose resin was visualized (Fig. 7C, D), as well as those with phosphatidylserine (Fig. 7E, F). The results suggested that the selected fluorescent proteins were effective for naked-eye observation of the affinity purification process.

### Solubilization efficiency of all proteins used in this study

**Fig. 8:**
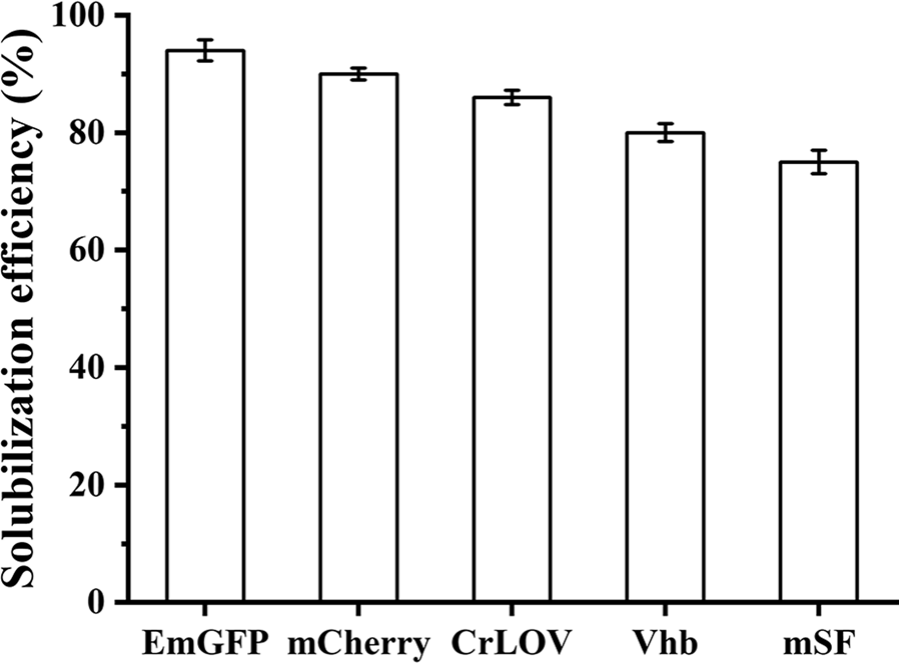
Solubility efficiency of three fluorescent proteins and two colored proteins. The solubilzation efficiency was calculated for protein amounts solubilized with EDTA-Na_2_ divided by protein amounts solubilized with urea

After separation of all proteins selected in this work, we tested the solubilization efficiency with EDTA-Na_2_ from the precipitated proteins with Ca^2+^ supplementation. Among the colored proteins, the EmGFP was most soluble (Fig. 8), due to the mutated several amino acid residues contributing the protein folding and solubility [28]. The mCherry was slight less solubilization efficiency than the EmGFP, but more than other proteins, attributed to the several changed residues contribution to the protein folding efficiency [31]. The solubilization efficiency of the CrLOV was less than other two fluorescent proteins. The CrLOV containing 118 amino acid residues is well-folded in *E. coli* [27]. The Vhb is solubly produced in *E. coli* [22], but about 15% the tagged wild type Vhb via CaCl_2_ precipitation was unable to be solubilized with the chelating reagent, possibly ascribed to the less folding efficiency. The mSF exhibited the least solubilization efficiency, most like resulted from the limited folding efficiency of the plant protein in *E. coli*. The results suggested that solubilization efficiency was related with hydrophobicity and inherent folding of the colored proteins.

## Discussion

In this study, we investigated the hanA1 as the N-terminal tag for rapid and cheap separation of the chosen proteins. The hanA1 aggregated in the presence of CaCl_2_ at specified concentrations most likely depended on its hydrophobicity. Presence of 100 mM NaCl in Tris–HCl buffer possibly increased the tag solubility, and/or decreased exposure of hydrophobic surface. This deduction is also explained that added CaCl_2_ displayed little impact on precipitating the purified His6-tagged hanA1-EmGFP in the diluted eluent containing 25 mM imidazole and 30 mM NaCl. Separation of the fusion protein was successful at 4 °C. Other fusion tags such as the ELP, hydrophobin and CspB are used for non-chromatographic purification under room temperature [6, 32, 33]. Different from the intein in the annexin fusion to potentially limit protein solubility [6], and the CSQ unable to be removed from the ZZ-CSQ [7], our constructs will be used for yielding the non-tagged target proteins via specific protease for cleaving the sequence introduced between the hanA1 and target protein, similar to the other proteases recognizing sequences incorporated between the ELP tag and target protein [34].

After two rounds of the separation processes, the purity and recovery of the hanA1-EmGFP were estimated about 75% and 67%, respectively (Table 1), less than about 95% recovery of the ZZ-CSQ [7]. Recovery of the His6-eGFP in *E. coli* with Ni–NTA purification is up to about 63%. Incorporation of the solubility enhancer Fh8 between the His6-tag and eGFP allowed the chimeric protein in clear lysate to be bound to phenyl Sepharose and protein recovery is about 77% [35]. In our study, elution of the extracted and separated hanA1-EmGFP from the same HIC afforded purified proteins with recovery of 78% and 71% (Table 3). The ELP-intein GFP overexpressed in *E. coli* with the optimized separation exhibits the recovery of about 53%, but the high purity [36]. Fusion of C-terminal silica-binding tag to the GFP renders purified fusion protein with the recovery of 78%, similar to the 76% recovery of the GFP-His6 [37], comparable with the separation efficiency of the hanA1-EmGFP. The Fh8 tagged GFP is bound to phenyl sepharose in a Ca^2+^-dependent manner [35]. In this work, the hanA1-EmGFP was eluted from the same resin by 10% saturated AS in the presence of 1 mM CaCl_2_. As comparison, the untagged GFP is strongly bound to phenyl sepharose, and eluted by the used buffer (20 mM Tris–HCl, pH 8.0, 1 mM EDTA) [38]. Our results suggested that fusion of the hanA1 changed the HIC property of the GFP, similar to that of the Fh8 tag.

The GFP is firstly identified for visual inspection of protein affinity purification process based on the application of affinity-fusion tags [39]. Our previous study visually tracked protein molecules released from the RAC using two fluorescent proteins and two colored proteins fused to the CBM tag [25]. In this work, we added the CrLOV for detecting the separation process. The protein precipitation and resolubilization were witnessed under UV irradiation and/or visible light, except for mSF with the Fe-S cluster disrupted by the added chelating reagent. The β-barrel based fluorescent protein variants such as EmGFP and mCherry protein are resistant to the presence of SDS, and the fluorescence on the SDS-PAGE gel is observed [40]. Here, we also perceived the CrLOV fluorescence on the SDS-PAGE gel. Recently, use of the LOV for detecting the specific ion is reported [41]. Our work indicated this fluorescent reporter presented visual display of separation of hanA1-CrLOV at relative high concentration. In-gel fluorescence provides the supplement technique for detection of other processes, such as in vitro cleavage of the fusion protein for the CrLOV with the specific protease, like our former reports [25, 26]. It is pointed out that not all fluorescent proteins are well folded in *E. coli*. Soluble production of the bilin-binding fluorescent protein like UnaG-bilirubin as a noncovalent ligand-dependent reporter requires fusion of the solubility enhancer GST or MBP [42, 43].

Unlike ELP, hydrophobin and CspB, the hanA1 were also absorbed with other affinity resins. In the current study, we firstly proved that the hanA1 was the new heparin-binding affinity tag, identical to the designed peptide [44]. The S100A1 via oriented immobilization was not worked well for purifying the hanA1-EmGFP. The CaM interacting protein fused with the MBP for amylose resin immobilization is well applied for purification of the CaM tagged GFP with the dissociation constant K_d_ to be 131.0 ± 38.6 nM [45]. The K_d_ for the short N-terminal peptide of human annexin I to Ca^2+^-S100A11 is found to be 5 ± 1 μM [13]. The less association reflected the immobilized S100A11 in this work with low binding capacity of the hanA1-EmGFP. Covalent coupling of the S100A11 and selection of the other member of annexin will be expected to increased purification efficiency. The same strategy will be also exploited for phosphatidylserine covalently coupled to the activated resin [14]. The lifetime of the expensive affinity matrix will be prolonged by combination of the separation and purification. Direct affinity purification avoid partial loss of the precipitated protein unable to re-solubilize with EDTA-Na_2_, as identification of solubility efficiency test.

The presence of 15 mM EDTA-Na_2_ affected the mSF structure through disrupting the Fe-S cluster. This compound also inhibits activities of metal-dependent enzymes. As far as we know, among the affinity tags, ceramic fluorapatite-binding peptides is used for purification of the fusion proteins through elution of bound proteins from the affinity matrix with high concentration of NaCl [46]. Low concentrations of two different compounds for eluting the MBP and GST tags from correspondent affinity matrices show relatively less impact on enzyme activity. In these cases, activity of the eluted protein or enzyme is analyzed after dilution of the eluent. Nonetheless, precipitation of proteins by means of suitable agents is an interesting alternative to state-of-the-art affinity chromatography [47], as usefulness of the attached hanA1 for controlling separation corroborated in this work. The separating and chromatographic properties of the hanA1 were preserved after it was fused to the colored proteins, and separation and purification of the fusion proteins were finished under mild conditions. Except for the mSF, other colored proteins are not affected by the redissolution method.

Generally, not any affinity tag is versatile for purifying the target protein, even with the His6-tag [48]. The *E. coli* contaminated protein is often co-eluted with the His6-tagged protein from the affinity resin [49]. The advantages and disadvantages of the commonly used affinity tags are described [1], and compared [35]. Removal of impurities during the precipitation/resolubilization process requires high speed centrifugation. For the ELP and hydrophobin tags, the expensive temperature-dependent centrifugation steps are required [32, 33]. As a comparison, addition the inexpensive affinity resins, such as microcrystalline cellulose or RAC to the clear lysate for absorbing the CBM tagged protein can be separated from the unbound proteins by relatively low speed centrifugation at room temperature. The hanA1 as the N-terminal appendix for various separation and purification handle offers great control of high selectivity in a single tag. The other affinity peptide, e.g. His6-tag and Strep-tag II, fused either to the hanA1 at N-terminus for forming the tandem affinity tag, or with the target protein at C-terminus to generate double affinity tag will further offer various purification strategies to obtain pure protein. The fluorescent and colored proteins selected in this study are ideal for testing the other tag applicability for Ca^2+^ dependent separation or purification, such as CSQ [7], and Fh8 tag.

## Conclusions

The findings presented here that the hanA1 was a new tag for separation and purification handle, as detected and witnessed by the selected five colored proteins. This fusion tag is potentially used for simple and cheap separation of soluble target protein with industrial or clinical value.

## Supplementary Information

**Additional file 1: Figure S1.** The synthetic gene encoding the CrLOV codon variant. **Figure S2.** SDS-PAGE analysis of the hanA1-EmGFP via two rounds of separation process. **Figure S3.** SDS-PAGE and Western blot analyses of the MBP-S100A11. **Figure S4.** Immobilization of the CBM-hS100A11 on RAC for binding and purifying the hanA1-EmGFP. **Figure S5.** Precipitation of the diluted eluent of purified His6-tagged hanA1-GFP from Ni–NTA resin with CaCl_2_ at different concentrations.

## Data Availability

The authors confirm that the data supporting the findings of this study are available within the article and its supplementary materials.
