# Supplementary material for: Detection of human annexin A1 as the novel N-terminal tag for separation and purification handle
Source: Microb Cell Fact. 2023 Jan 5;22:2. doi: 10.1186/s12934-022-02005-x (PMC9817314; doi:10.1186/s12934-022-02005-x)
Supplement: Supplementary file 1 — Additional file 1: Figure S1. The synthetic gene encoding the CrLOV codon variant. Figure S2. SDS-PAGE analysis of the hanA1-EmGFP via two rounds of separation process. Figure S3. SDS-PAGE and Western blot analyses of the MBP-S100A11. Figure S4. Immobilization of the CBM-hS100A11 on RAC for binding and purifying the hanA1-EmGFP. Figure S5. Precipitation of the diluted eluent of purified His6-tagged hanA1-GFP from Ni–NTA resin with CaCl2 at different concentrations. [file 12934_2022_2005_MOESM1_ESM.docx]

Detection of human annexin A1 as the novel N-terminal tag for separation and purification handle

Xiaomei He^1,2^, Shuncheng Zhang^2^, Dongya Dang^2^, Tingting Lin^2^, Yuanyuan Ge^2^, Xiaofeng Chen^2^, Jun Fan^2*^

1: College of Biology and Pharmaceutical Engineering, West Anhui University, Lu'an, 237012, PR. China.

2: School of Life Science, Anhui Agricultural University, Hefei, Anhui, 230036, PR China.

*Corresponding author. Jun Fan. E-mail address: fanjun@ahau.edu.cn. Postal address: School of Life Science, Anhui Agricultural University, 130, Changjiang West Road, Hefei, 230036, Anhui Province, PR. China.

*Bam*HI

*ggatcc*gataaaataaaagcaggtctgcgccatacctttgtggtggcggatgcgaccctgccg

G S D K I K A G L R H T F V V A D A T L P

gattgcccgctggtgtatgcgagcgaaggcttttatgcgatgaccggctatggcccggat

D C P L V Y A S E G F Y A M T G Y G P D

gaagtgctgggccataacgcgcgctttctgcagggcgaaggcaccgatccgaaagaagtg

E V L G H N A R F L Q G E G T D P K E V

cagaaaattcgcgatgcgattaaaaaaggcgaagcgtgcagcgtgcgcctgctgaactat

Q K I R D A I K K G E A C S V R L L N Y

cgcaaagatggcaccccgttttggaacctgctgaccgtgaccccgattaaaaccccggat

R K D G T P F W N L L T V T P I K T P D

ggccgcgtgagcaaatttgtgggcgtgcaggtggatgtgaccagcaaaaccgaaggcaaa

G R V S K F V G V Q V D V T S K T E G K

*Xho* I

Gcgctggcgagctaa*ctcgag*

A L A S -

Figure S1 The synthetic gene encoding the CrLOV codon variant. The fragment with excision of *Bam*H I and *Xho* I was inserted into the pA-EmGFP plasmid for expressing the hanA1-CrLOV.


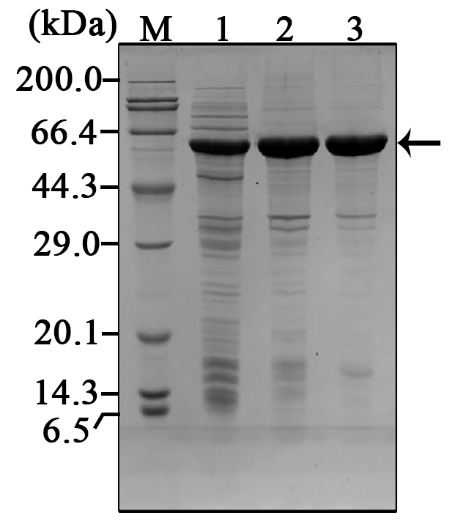


Figure S2 SDS-PAGE analysis of the hanA1-EmGFP via two rounds of separation process. M: protein marker. Lane 1: overexpressed proteins in the supernatant. Lane 2: supernatant via first Ca^2+^ precipitation and EDTA-Na_2_ resolubilization. Lane 3: the separated hanA1-EmGFP was further separated through second Ca^2+^ precipitation and EDTA-Na_2_ resolubilization. The arrow indicated the bands representing the overexpressed fusion protein.


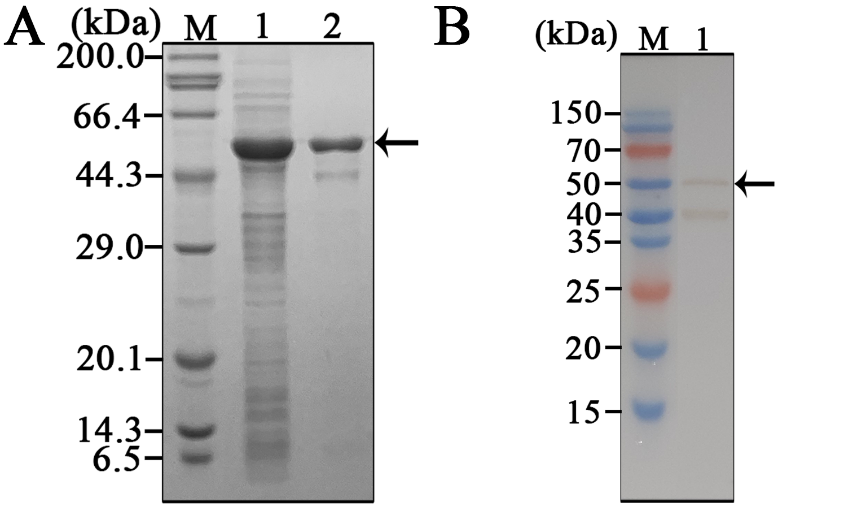


Figure S3 SDS-PAGE and Western blot analyses of the MBP-S100A11. (A) Overexpression of the fusion protein and immobilization of the fusion protein on the amylose resin. Lane 1: overexpressed proteins in the supernatant. Lane 2: the immobilized protein after washing with 50 mM Tirs-HCl, pH7.0 containing 200 mM NaCl. (B) Western blot analysis of the co-eluted MBP-S100A11 for purifying the hanA1-EmGFP. Lane 1: the eluent was detected by anti-MBP antibodies. Arrows indicated the MBP-S100A11.


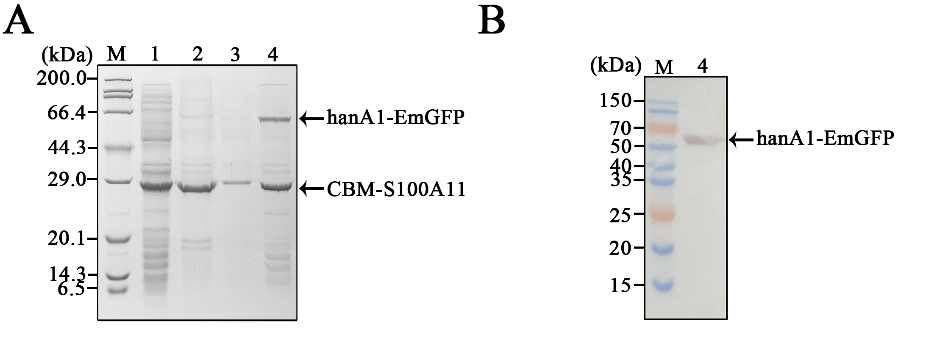


Figure S4 Immobilization of the hS100A11 fused with the CBM tag on the RAC for binding and purifying the hanA1-EmGFP. A: SDS-PAGE analysis of the hanA1-EmGFP eluted from the hS100A11 via oriented immobilization. Lane 1: soluble extracts from the overexpressed CBM-S100A11. Lane 2: the bound proteins after washing with buffer containing 500 mM NaCl. Lane 3: the washed proteins from the immobilized S100A11 interacting with the hanA1-EmGFP in crude extract. Lane 4: the proteins from the resin eluted with buffer C. B: Western blot analysis of the eluted protein sample using anti-GFP antibodies. Lane 4 in SDS-PAGE and Western blot analyses were identical.


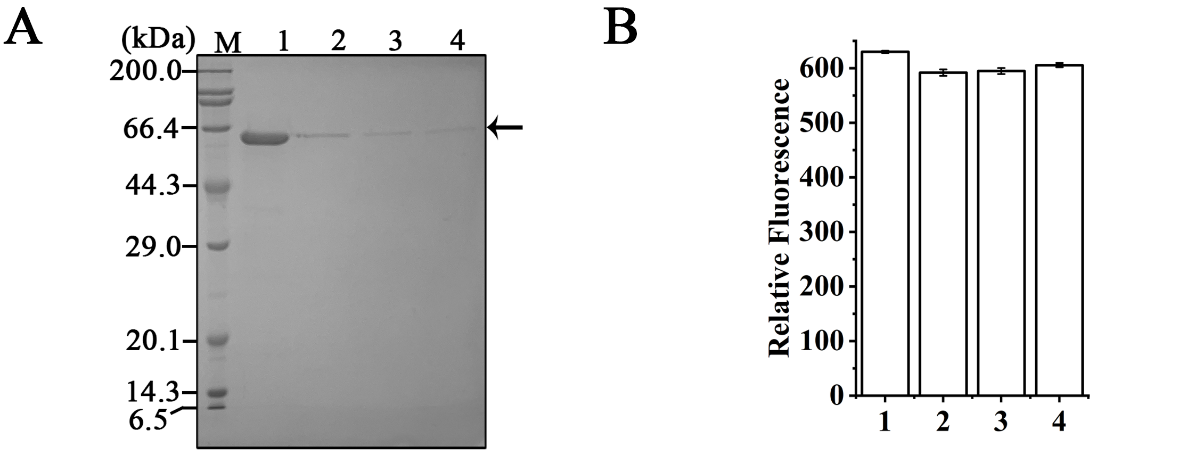


Figure S5 Precipitation with CaCl_2_ at different concentrations. (A) SDS-PAGE analysis of the added CaCl_2_ at final concentrations of 20, 50 and 100 mM to precipitate protein in the eluent diluted with buffer A for ten folds. Lane 1: purified fusion protein with addition of buffer A as control. Lanes 2-4 samples were precipitate with use of 20, 50 and 100 mM CaCl_2_, respectively. (B) The retained fluorescence from the supernatant after precipitation. All arrows indicated the His6-tagged hanA1-GFP. 1: the fluorescence from purified proteins with dilution ten folds. 2-4: the retained fluorescence from the supernatant after the supplemented CaCl_2_ precipitation. Data were from three technical replicates (average ± standard deviation).
